# Functional, Cellular, and Molecular Remodeling of the Heart under Influence of Oxidative Cigarette Tobacco Smoke

**DOI:** 10.1155/2017/3759186

**Published:** 2017-07-20

**Authors:** Abdullah Kaplan, Emna Abidi, Rana Ghali, George W. Booz, Firas Kobeissy, Fouad A. Zouein

**Affiliations:** ^1^Department of Pharmacology and Toxicology, American University of Beirut Faculty of Medicine, Beirut, Lebanon; ^2^Department of Pharmacology and Toxicology, University of Mississippi Medical Center School of Medicine, Jackson, MS, USA; ^3^Department of Biochemistry and Molecular Genetics, American University of Beirut Faculty of Medicine, Beirut, Lebanon

## Abstract

Passive and active chronic cigarette smoking (CS) remains an international epidemic and a key risk factor for cardiovascular disease (CVD) development. CS-induced cardiac damage is divided into two major and interchangeable mechanisms: (1) direct adverse effects on the myocardium causing smoking cardiomyopathy and (2) indirect effects on the myocardium by fueling comorbidities such as atherosclerotic syndromes and hypertension that eventually damage and remodel the heart. To date, our understanding of cardiac remodeling following acute and chronic smoking exposure is not well elucidated. This manuscript presents for the first time the RIMD (oxidative stress (R), inflammation (I), metabolic impairment (M), and cell death (D)) detrimental cycle concept as a major player in CS-induced CVD risks and direct cardiac injury. Breakthroughs and latest findings in the field with respect to structural, functional, cellular, and molecular cardiac remodeling following chronic smoking exposure are summarized. This review also touches the genetics/epigenetics of smoking as well as the smoker's paradox and highlights the most currently prominent pharmacological venues to mitigate CS-induced adverse cardiac remodeling.

## 1. Introduction

Cardiovascular diseases (CVDs) remain the leading cause of morbidity and mortality worldwide. The World Health Organization (WHO) estimates a total of 17.5 million annual CVD deaths (31% of total death) mostly due to myocardial infarction (MI) and strokes (http://www.who.int/cardiovascular_diseases/en/). Tobacco smoke is a major risk factor for CVDs, and chronic tobacco smoking (CS) increases the risk of coronary artery disease (CAD) by two- to fourfold [1, 2]. WHO estimates a total of 5 million deaths yearly due directly to tobacco smoke and more than 600,000 deaths due to second-hand smoke. People who are exposed to second-hand smoke are at a 25–30% risk of developing heart disease and 20–30% risk of stroke [1]. Tobacco smoke contains more than 4720 compounds including well-known harmful chemicals such as polycyclic aromatic hydrocarbons, free radicals, and oxidative gases [3, 4]. Although multiple studies show that oxidative compounds are primarily responsible of smoking-mediated myocardial injury [3, 5–9], the exact mechanisms of numerous potentially harmful smoke compounds remain poorly understood and require further investigation. This review highlights the effect of CS on structural, functional, and molecular cardiac remodeling based on most recent breakthroughs and latest publications. Promising pharmacological interventions that protect the heart from the cigarette smoking epidemic are summarized in this manuscript.

## 2. Cardiac Remodeling Post-CS: Structural and Functional Level

Cardiac remodeling occurs in response to both physiological and pathological stimuli including exercise, myocardial infarction, arterial hypertension, valvular diseases, and myocarditis. In pathological response, both molecular and cellular changes can lead to either ventricular hypertrophy and/or dilatation that are functionally translated into diastolic and/or systolic dysfunction. The term smoke cardiomyopathy was first introduced by Gvozdjakova et al. to refer to metabolic and morphological alterations in the rabbit myocardium after chronic CS and in the absence of comorbidities [10, 11]. Numerous studies highlight the negative impact of acute and chronic smoking on ventricular systolic and diastolic function in human and rodents [12–18]. Direct and indirect toxic effects to the myocardium have been reported with CS exposure both clinically and experimentally [19]. Direct effects such as myocardial ischemia, necrosis and fibrosis, focal myocardial hemorrhage, focal myocarditis, myocardial fiber swelling, functional and structural alteration of myocardial mitochondria, coronary vasocontraction, and cardiac arrhythmias have been documented [19]. CS indirect effects on the heart are attributed to blood pressure increase, plasma cholesterol level alteration, increased plasma viscosity and platelet aggregations, T-cell function alteration, and increased inflammatory profile [19]. CS-mediated increase in blood pressure and heart rate was attributed to sympathetic outflow enhancement to blood vessels and heart, respectively [20]. However CS-induced sympathetic effects on the heart did not increase cardiac inotrope mainly due to CS-mediated myocardial oxygen deficiency due to carbon monoxide and carboxy hemoglobin accumulation [21]. Cardiac hemodynamic changes in rodents exposed to smoke with different exposure times are documented. Mice exposed to 32 weeks of smoke showed impaired systolic and diastolic function when left ventricular (LV) pressure-volume relationship was assessed at high afterloads [22]. Dawley rats exposed to 5 weeks of smoke exhibited a significant increase in LVEDD and LVESD along with a significant decrease in FS when compared to the control group [23]. In addition to systolic dysfunction and increased left ventricular systolic volume, rats exposed to 8 weeks of CS experienced an enlargement of the left atrium [24].  Table 1 summarizes a selective list of preclinical CS studies and their impact on the structure and function of the heart in the presence or the absence of comorbidities such as MI or volume overload.

## 3. Cardiac Remodeling Post-CS: Cellular and Molecular Level

**Figure 1:**
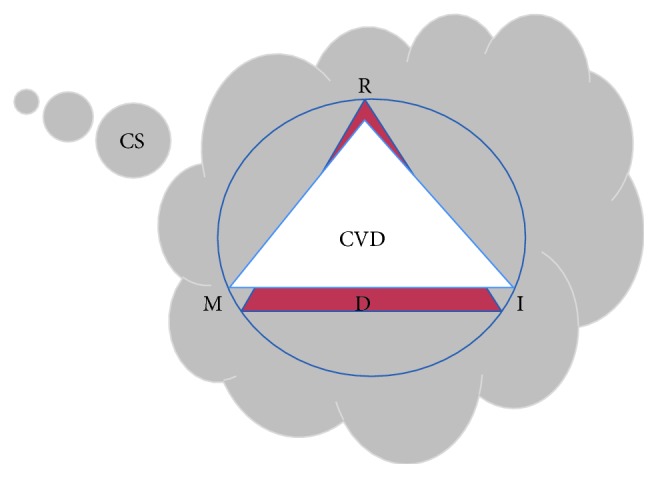
Vicious and detrimental cycle of CS-induced cardiovascular injury. In addition to exogenous ROS delivery, chronic smoking exposure attenuates antioxidant defenses and increases endogenous ROS formation. Oxidative stress (R) will eventually trigger an inflammatory response (I) and metabolic impairment (M) and subsequent cell death (D) that fuel ROS formation. This chronic phenomenon could lead to CVD. The presence of CVD exacerbates CS-mediated RMID process detrimental effects.

Myocardium cellular and molecular impairment following CS is directly related to at least 4 interchangeable mechanisms that are termed RIMD in this review and include oxidative stress (R), inflammation (I), metabolic impairment (M), and cell death (D) [3, 9, 11, 25, 26]. In response, matrix metalloproteinase (MMP) activation, mitogen-activated protein kinase (MAPK) activation, mitochondrial dysfunction, lipotoxicity, neurohormonal imbalance, gap junction remodeling, immune cell infiltration, and other events contribute to the adverse remodeling of the myocardium [3, 9, 23, 24, 27–30]. Direct adverse effects of CS on myocardium are exacerbated by their general effects on the cardiovascular system, creating a vicious adverse remodeling cycle that can acutely or progressively damage the heart (Figure 1). In fact, CS is directly responsible for several clinical atherosclerotic syndromes, including stable and unstable angina, aortic atherosclerosis, coronary lesion, and sudden death [31–34]. A large number of studies support the direct stimulatory effect of CS on multiple components of atherosclerotic syndrome progression including, endothelial/vasomotor dysfunction [35–42], dyslipidemia [43–46], inflammation [47–50], platelet dysfunction [51–53], and alteration in antithrombotic, prothrombotic, and fibrinolysis factors [54–57].

### 3.1. Oxidative Stress

Oxidative stress occurs as an imbalance between reactive oxygen species (ROS) production and antioxidant defense mechanisms within the cell. Under physiologic conditions, ROS production occurs as result of oxygen metabolism and plays a vital role in cell signaling [58, 59]. However, unbalanced and excessive ROS production in response to exogenous and endogenous stimuli can cause lipid peroxidation, DNA strand breaks, and other forms of intracellular injury which are detrimental to cellular structure and function [58]. In addition to intracellular ROS induction, cigarette smoke contains substantial amounts of ROS and other chemicals that diminish the intracellular antioxidant mechanisms [5, 9]. As a result, inflammation, MMP activation, cardiac fibroblast proliferation, and intracellular remodeling pathway stimulation contribute to CS-induced cardiac remodeling [9, 60]. Talukder et al. showed that both mononuclear and polymorphonuclear blood cells from 32 week CS-exposed mice generated more H_2_O_2_ and superoxide than controls leading to systemic oxidative stress and mild cardiac hypertrophy in mice that were not otherwise predisposed to disease [22]. Santos et al. reported similar pathology in rats exposed to 8 weeks of CS. Their analysis revealed direct cardiotoxicity characterized by decreased glutathione peroxidase and superoxide dismutase activity, decreased fatty acid (FA) oxidation, and subsequent increase in ROS formation, lipotoxicity (increased lipid peroxidation), and mitochondrial dysfunction [24]. Duarte et al. assessed the impact of CS on ventricular remodeling following myocardial infarction. CS exacerbated LV remodeling post-MI with a significant increase in glutathione disulfide (GSSG) and a decreased glutathione (GSH), GSH/GSSG ratio in both the heart and the liver [25]. Those findings suggest an impaired systemic antioxidant defense and further support the systemic oxidative impact of CS on cardiac remodeling. Multiple studies support the systemic oxidation impact using drugs with antioxidant properties to rescue CS-induced cardiac remodeling. Those rescue-type therapies are discussed in the pharmacological venue section of this review.

### 3.2. Inflammation

Regardless of the underlying mechanisms, cardiac remodeling is often accompanied by an inflammatory response. Several clinical and preclinical studies positively correlate heart failure with high levels of proinflammatory cytokines [61–66], and an inflammatory response post-CS exposure has been well documented. In the case of smoking, combustion can trigger the production of ROS that are normally not present in the leaf or the ash [67] but are enriched in both the gaseous and particulate components of smoking [68, 69]. Such alteration in ROS production due to cigarette smoking has drastic effect on the host immunity affecting the innate immunity in the airway mucosa, as well as adaptive immunity at the systemic level [70, 71]. ROS play a crucial role in the inflammation process by initiating a variety of ROS-sensitive signaling pathways including mitogen-activated protein kinases (MAPKs) and a number of transcription factors, such as nuclear factor-κB (NF-κB). Subsequently, inflammatory gene expression is triggered increasing the production of proinflammatory cytokines such as interleukin-8 (IL-8) and tumor necrosis factor-alpha (TNF-*α*) [70, 72]. The secretion of these inflammatory cytokines leads to chronic immune cell recruitment and inflammation [73]. Walters and colleagues demonstrated for the first time that smoke stimulates IL-8 release from monocytes and macrophages in an oxidant-mediated phenomenon and mainly by acting via activator protein-1 (AP-1) pathway along with tonic levels of NF-κB activation [73]. Walters work also revealed that inhibition of IL-8 release is steroid resistant only when stimulation is primed with IL-1*β*. Those findings could elucidate the mechanisms behind clinical, smoking-induced, inflammatory response resistance to glucocorticoid treatment [74]. Although oxidative stress and inflammation are normally coassociated together, Das et al. demonstrated that inflammatory response follows oxidative stress in a two-week margin following CS in pigs [9]. Oxidative damage was detected after two weeks of CS exposure while proinflammatory cytokines including TNF-*α* and IL-1*β* were detected after 4 to 6 weeks of CS. Neutrophil infiltration into the myocardium was observed around 6 to 8 weeks following CS [9]. These studies highlight the effect of oxidative damage in releasing danger associated molecular patterns (DAMPs) that trigger an immune cell response and subsequent inflammation [75]. The inflammatory profile was assessed by Zhou et al. in two independent studies following 4 months of CS exposure in rats. Their findings revealed an enhanced cardiac gene expression of proinflammatory cytokines including IL-1*β*, IL-6, and TNF-*α* and serum circulating levels of Hs-CRP, IL-6, TNF-*α*, IL-1*β*, and MCP [60, 76] suggesting systemic inflammation. Other studies supported both the upregulation of other proinflammatory cytokines including IL-2, IL-8, IFN-*γ*, and GM-CSF and the downregulation of anti-inflammatory cytokines such as IL-10 [77, 78]. However, other studies reported controversial results. Duarte et al. and Santos et al. observed that CS did not impact myocardial levels of multiple cytokines inducing IFN-*γ*, TNF-*α*, IL-10, and ICAM-1 [28, 79]. One explanation for these contradicting findings could be the inconsistencies between studies as far as CS exposure time and concentration.  Table 1 highlights different findings with respect to CS exposure protocols.

### 3.3. Metabolic Impairment

**Figure 2:**
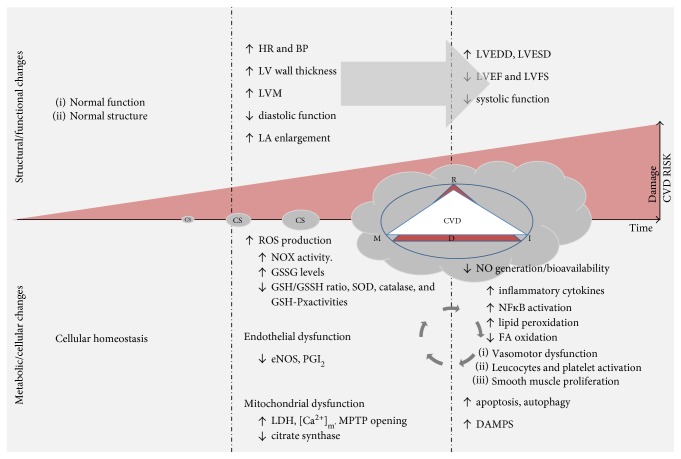
The impact of chronic smoking exposure on CVD risk and cardiac structural, functional, and cellular damage. CS-based RMID cause metabolic and cellular damages that alter cardiac structure and function and increase CVD risk and myocardial damage. HR, heart rate; BP, blood pressure; LV, left ventricle; LVM, left ventricular mass; LA, left atria; LVEDD, left ventricular end-diastolic diameter; LVESD, left ventricular end-systolic diameter; LVEDV, left ventricular end-diastolic volume; LVEF, left ventricular ejection fraction; LVFS, left ventricular fractional shortening; ROS, reactive oxygen species; NOX, NADPH oxidase; GSSG, glutathione disulfide; GSH/GSSH, glutathione disulfide/glutathione ratio; SOD, superoxide dismutase; GSH-px, glutathione peroxidase; eNOS, endothelial nitric oxide synthases; PGI_2_, prostacyclin; LDH, lactate dehydrogenase; [Ca^2+^]_m_, mitochondrial calcium; MPT, mitochondrial permeability transition; NF-κb, nuclear factor-κB; FA, fatty acid; DAMPs, danger-associated molecular patterns.

Cellular metabolic impairment constitutes another source of oxidative stress that fuels oxidative damage and subsequent inflammatory response post-CS. Low ROS levels are normally generated during oxidative phosphorylation in mitochondria [80] and kept within a normal range due to the presence of antioxidant mechanisms like glutathione, vitamin E, catalases, peroxidases, and superoxide dismutase (SOD) [81]. Under normal conditions, oxidants regulate intracellular processes, but excess oxidants induce irreversible damage to cellular components leading to altered cellular functions or apoptosis [82]. In vitro, ROS were shown to cause mitochondrial injury by attenuating mtDNA-encoded mRNA transcripts, altering mitochondrial protein synthesis and decreasing cellular ATP levels and mitochondrial redox potential [83]. By virtue of their importance in cell signaling and apoptosis, mitochondria may modify cellular energy production and regulatory pathways in cardiovascular cells [84]. Mitochondrial signaling disruption due to chronic oxidant exposure could thereby lead to reduced energy production and induce cell death mainly in tissues with low energetic thresholds [84]. Morphological and functional changes in myocardial mitochondria including swelling, external membrane alteration, lipid accumulation, oxidative phosphorylation rate reduction, ROS production, and mitochondrial permeability transition pore (mPTP) opening have all been reported in animals following CS [11, 26, 85–87]. Gvozdjakova et al. linked the “smoke cardiomyopathy” mainly to mitochondrial dysfunction [11, 26]. They showed a significant decrease in respiration as well as in the phosphorylation rate of mitochondria, diminished cytochrome oxidase activity, and decreased coenzyme Q10 (CoQ10) levels, three weeks following passive CS exposure in rabbits. CoQ10 is a critical component of normal mitochondrial ETC function and plays an important role in reducing lipid peroxidation and ROS scavenging [88]. Santos et al. reported, in their 2-month CS exposure study in rats, abnormalities in energy metabolism, including lipotoxicity and oxidative stress [24]. This study revealed impaired mitochondrial respiration through enhanced lactate dehydrogenase activity and decreased citrate synthase activity, and an impaired FA oxidation as evidenced by reduction in 3-hydroxyacyl coenzyme-A dehydrogenase level and increased triglyceride density in cardiac tissue [24]. Last but not least, Yamada et al. concluded that clinically relevant concentration of aqueous extract of cigarette smoke increases cardiomyocyte mitochondrial Ca^2+^ load during simulated ischemia and the susceptibility of mPTP opening and subsequent cell death. These findings were partly attributed to tobacco smoke-induced ROS [89]. In summary, metabolic impairment post-CS exposure plays a major role in cellular dysfunction by participating in the vicious cycle of CS-induced RIMD (Figure 2).

### 3.4. Cell Death

ROS, inflammation, and metabolic impairment post-CS exposure predispose cardiac cells to death [9, 24, 30, 60, 90]. In their vitamin C-deficient guinea pig model, Das et al. documented the induction of both intrinsic (cytochrome C release, p53 phosphorylation, increased Bax/Bcl-2 ratio, and activation of caspase 3) and extrinsic (TNF-*α* upregulation and caspase 8 activation) pathways of apoptosis in the myocardium in a time-dependent manner after CS exposure [9]. High cardiomyocyte apoptotic rate was also shown via annexin V/PI staining by Zhou et al. in a CS rat model [60]. On a separate study, Zhou et al. reported the induction of JNK and P38 of MAPK signaling pathways and the inhibition of PI3K/AKT pathways in the myocardium following CS exposure to rats [30]. Both pathways are known to be involved in cellular apoptosis [91, 92]. Increased extracellular signal-regulated kinase (ERK1/2) and p38 of MAPK were also associated with CS-induced left ventricular remodeling [23]. In addition to apoptosis, autophagy was also reported to play a pathogenic role in smoking-induced left ventricular systolic dysfunction [30, 93].

### 3.5. Genetic and Epigenetics of Smoking-Induced CVD

Genetic and epigenetic predisposition to smoking-induced CVD have been established but barely elucidated. One renowned genetic example is the interaction of apolipoprotein E (ApoE) variants with smoking. ApoE isoform, *ϵ*4, is associated with increased oxidized lipoprotein production and subsequent CVD risks in the presence of smoking-induced excessive ROS [94, 95]. Sequence variants in multiple other proteins including TGF-*β*, lipoprotein lipase, IL-18, IL-6, and prothrombin have been involved in smoking-related CVD risks [94, 96–98]. In addition to genetic variation, epigenetics of CVD gained significant attention in recent years. In the context of tobacco-smoking, few epigenetic patterns associated with CVD have been recognized [99, 100]. Recent epigenome wide studies identified a significant link between coagulation factor II receptor-like 3 (F2RL3) lower methylation and smoking behavior [99, 101]. F2RL3 gene encodes for protease-activated receptor 4 (PAR4), a protein that highly correlates with multiple cardiovascular pathophysiological events including inflammation, platelet function, myocardial injury, and death [101–104]. Other studies highlighted the association of smoking with alteration of microRNA expression in spermatozoa and subsequent potential adverse outcomes in offspring [105]. Evidence of smoking interaction with a flavin-containing monooxygenase 4 (FMO4) variant has also been reported with respect to coronary heart disease (CHD) [106]. Identifying genetic and epigenetic association with smoking-induced CVD is a promising tool to improve cardiovascular risk prediction, personalized prevention, and intervention in smokers. However, to date, gene-smoking interaction and pattern discoveries remain highly novel, multifactorial, and technically and statistically very challenging.

## 4. The Smoker's Paradox

Although history of smoking highly correlates with adverse cardiovascular risk, an old-new phenomenon known as “smoker's paradox” emerged, describing the potential beneficial effects of smoking on the cardiovascular system. The smoker's paradox was first noted in 1977 on a group of acute MI patients and later confirmed in multiple clinical cardiovascular studies including fibrinolysis, acute stroke, cardiac arrest, and ST elevated MI (STEMI) [107–111]. Multiple preclinical studies confirmed the paradoxical effect of smoke on mortality after MI and attributed the beneficial effects to cardiac gap junction remodeling and preconditioning [27, 112]. Smoking was independently associated with improved survival in patients with cardiac arrest and lower inpatient mortality in acute ischemic stroke and STEMI [107, 110, 111]. Results were attributed in part to changes in vascular reactivity due to a global smoking-induced ischemic conditioning. On average, compared with nonsmokers, smokers are consistently younger on hospital admission with fewer comorbidities and better overall prognosis. Nonetheless, GUSTO-1 trial revealed a 25% lower mortality rate in smokers 30 days post-AMI thrombolysis, even after adjusting for age, sex, blood pressure, and other covariates [108]. In contrast, recent SYNTAX trial findings revealed that smoking was associated with a higher rate of death/MI/stroke (86% MI hazard) at 5 years follow-up following coronary artery bypass grafting (CABG) and percutaneous coronary intervention (PCI) for stable CAD-treated smoker patients [113]. SYNTAX recommended smoke cessation to improve revascularization benefits. The contradictory findings between GUSTO-1 and SYNTAX could be explained as follows: (1) the GUSTO-1 trial studied the impact of short-term (30 days) impact of smoke versus 6 months to 5 years for SYNTAX; (2) the GUSTO-1 trial focused on thrombolysis revascularization following AMI versus CABG and PCI in stable CAD for SYNTAX; and (3) SYNTAX allowed smokers who quit during trial to be analyzed as nonsmokers during the interval in which they quit [108, 113]. Multiple recent clinical studies denied CS protective effects and concluded that smoker's paradox is in fact a pseudoparadox [114–116]. Their findings revealed no relevance of smoking paradox among patients undergoing primary PCI [114]. Additionally, smoking history had no significant effects on infarct size 30 days post-MI [115]. In fact, a growing consensus support the hypothesis that smoker's paradox is largely attributed to differences in demographics and clinical baseline risks and that smoking survival benefit in univariable analysis is mostly related to younger population with low CV risks, shorter time exposure, and absence of comorbidities [116].

Clearly the long-known clinical controversy behind the paradox of smoking is shrinking. However, experimental evidence of CS-induced cardioprotection could be related to potential unknown bioactive protective cigarette compounds. Their discovery and isolation from harmful compounds could devise novel therapeutic strategies to improve the overall prognosis following acute cardiac events.

## 5. Pharmacological Venues

Smoking cessation is currently the most adapted strategy to treat smoking habit. A variety of smoking cessation therapies are clinically available and divided into two major groups: drug-based therapy and drug-free therapy [117]. Drug-based therapy involves nicotine replacement therapy (NRT) including nicotine gum and nicotine skin patches and prescription drug therapy (PDT) including nicotine receptor antagonists (bupropion hydrochloride) and nicotine receptor partial agonist (varenicline) [117, 118]. Drug-free therapy involves awareness and counseling to reduce smoking progressively and rapidly [119, 120]. However, smoking cessation compliance among smokers is limited due to drug side effects and/or socioeconomic constraints which increase the chances of relapse [121, 122]. In CVD, a novel therapeutic strategy, as adjunct to smoking cessation attempts, is to clinically focus on reducing the smoking adverse effects on the heart in both acute and chronic events such as smoking cardiomyopathy or AMI to improve the overall prognosis. Multiple preclinical studies have successfully documented the importance of multiple supplements and drugs in reducing the adverse effect of smoking on the heart (Table 2). For instance, Rafacho et al. revealed that vitamin D supplementation attenuates cardiac remodeling post-CS in rats by increasing superoxide dismutase and catalase activity and lowering lipid oxidation [123]. Das et al. reported that vitamin C supplementation to CS-exposed guinea pig prevented adverse cardiac remodeling post-MI mainly by reducing and inactivating p-benzoquinone (p-BQ), a redox cycling agent produced from p-BSQ of CS and a major factor that produces ROS, oxidative damage, and apoptosis [9, 123]. Reduced oxidative stress and improved cardiac bioenergetics were reported in pentoxifylline (nonselective phosphodiesterase (PDE) inhibitor)-treated rats that were exposed to CS [90]. In addition to ROS scavenging, attenuating inflammatory and apoptotic response post-CS exposure has also been reported [30, 60, 76, 78]. Multiple drugs including trimetazidine, N-acetyl cysteine, hydrogen sulfide (H2S), and valsartan reduced smoking-induced inflammation and/or apoptosis and, subsequently, modulated smoking-mediated left ventricular dysfunction [30, 60, 76, 78]. In conclusion, drugs that target the detrimental smoking-induced cycle of RIMD seem to exert beneficial effects by attenuating CS-induced cardiac dysfunction in the presence or absence of acute cardiac events. Of note, RIMD branches are dependent and interchangeable, meaning that any drug that affects one branch, such as ROS, could affect the concept as a whole. Those approaches should be further explored in the clinics with the attempt to increase the overall prognosis of chronic smokers by either preventing smoking-induced cardiomyopathy or improving cardiac remodeling following an acute cardiac injury such as MI.

## 6. Conclusions and Future Perspectives

Despite increased social awareness, marketing restraints, tobacco taxation, and available smoking cessation rehabs, active and passive smoking remains a worldwide challenging epidemic with high CVD risks and other fatal diseases. In this review, we presented for the first time the concept of RIMD to explain CS-mediated adverse effects on the myocardium. CS impact on the myocardium is multifactorial and directly related to 4 main interchangeable mechanisms including ROS generation, metabolic impairment, inflammation, and cell death. Chronic exposure to smoking induces molecular and cellular alteration that could translate into myocardial structural and functional changes that vary between individuals depending on their genetic and epigenetic predisposition. To date, no clinically effective therapy is adapted to reduce CS-induced adverse effects on the myocardium in the presence or absence of comorbidities. Alternative solutions to smoking cessation should be implemented. For instance, our current understanding of functional, structural, molecular, cellular, genetic, and epigenetics of smoking-associated cardiac remodeling should be escalated in order to devise novel therapeutic strategies that limit smoking-mediated CVD and associated comorbidities. Multiple promising supplements and drugs remain in the preclinical phase with no obvious signs of potential clinical adoption.

## Figures and Tables

**Table 1:** A selective list of studies highlighting the effect of CS on cardiac remodeling.

Model	Study design^¥^	Cardiac remodeling						Ref
		Structural/functional	Inflammation	Oxidative stress	Apoptosis	Metabolic impairment	Others	
C57BL/6J mice: *32 weeks of CS exposure*	CS effect on LV remodeling in mice	↑ SBP, DBP ↑ HW : BW ratio, LVM ↓ EDV and CO	N/A	↑ ROS in white blood cells	N/A	N/A	↑ NO decay ↑ ED	[22]
		(i) Impaired LV P-V relationship at high afterload						

S-D rats: *1 week CS exposure prior to abdominal aortocaval fistula surgery and for 6 weeks thereafter*	CS effect on LV remodeling in volume overloaded heart	↑ LV dilation ↓ LVPWT, eccentric index, FS ↔ HR, CO, BP	N/A	N/A	N/A	N/A	↓ collagen deposition ↓ ET-1, HIF1*α*, VEGF, TGF-*β* protein levels ↑ MMP-9, TIMP-1 protein levels ↔ MMP-2 protein levels	[29]

S-D rats: *5 weeks of CS exposure*	CS effect on LV remodeling in rats	↑ LVEDD, LVESD, E/A ↑ HW : BW ratio ↓ FS ↔ LVPWT	N/A	N/A	N/A	N/A	↑ urinary NE levels ↑ Pp38/total p38 ↑ PERK1/2/ERK1/2 ↔JNK levels	[23]

Rabbit-mouse VM exposed to 0.1% aqueous extract of cig smoke	Effects of aqueous extract of cigs on isolated VM	↑ ischemic injury ↑ myocyte contracture	N/A	↑ ROS	N/A	↑ susceptibility to mPTP opening	↑ myocyte mito [Ca2+]m uptake ↑ myocyte cytosolic [Ca2+]i during ischemia	[89]

Wistar rats: *20 cigs/day first week then 40 cigs/day for 4 weeks*	CS effect on cardiac Cx43	↔ LVW, RVW, CSA	N/A	N/A	N/A	N/A	↔ Cx43 distribution at intercalated disks ↓ Cx43 intensity at intercalated disks ↑ Cx43 dephosphorylation ↑ lateralization ↔ total Cx43 levels ↔ CVF	[27]

Wistar rats: *20 cigs/day first week then 40 cigs/day until 2 months*	CS effect on LV remodeling	↑ LA area, CSA ↑ LVSV ↓ EF, FS	N/A	↑ ROS ↓ SOD and GSHPx activities	↑ apoptosis	↓ OHDAH, CS activities ↑ LDH activity ↑ serum VLDL, LDL, TG, myocardial TG ↓ serum HDL	↔ PPAR-*α*, PGC-1*α*	[24]

Wistar rats: *6 months of CS exposure starting at 48 hours post-MI*	CS effect on LV remodeling post-MI	↑ HR, LA area, E/A, DA, SA ↑ RVW : BW ratio, LW	N/A	↑ GSH, GSSG ↓ GSH/GSSG ratio ↔ LOOHs levels	N/A	N/A	↔ CVF	[25]

C57BL/6 mice: *20 cigs/day for 1 month*	CS effect on LV remodeling	↑ BP, LVH, HW : BW ratio ↔ HR	↑ IL-6, TNF-*α* serum levels ↔ IL-6, TNF-*α*	N/A	N/A	N/A	↑ eNOS, iNOS, sGC*α*, sGC*β*, pVASP, cGMP ↑ mRNA *β*-MHC ↔ PKG, PDE5 ↓ H2S producing enzymes	[124]

C57BL/6 mice: *20 cigs/day for 1 month*	CS effect on LV remodeling followed by NoC, PreC or PostC I/R	↔ infarct size (NoC, PreC) ↑ infarct size (PostC)	N/A	↔ PC, MDA (NoC, PreC) ↑ PC, MDA (PostC)	N/A	N/A	↔ Akt, eNOS, cGMP activation (PreC) ↓ Akt, eNOS, cGMP activation (PostC) ↔ Akt, ↑ eNOS, and cGMP (NoC) ↓ pVASP (PostC and PreC) ↑ pVASP (NoC) ↓ nitrate + nitrite (PostC and PreC) ↔ nitrate + nitrite (NoC)	[124]

^¥^Results presented in this table are in comparison with nonsmoking same conditioning treatment. CS: chronic tobacco smoking; S-D: Sprague-Dawley; N/A: not available; ↑, increase; ↓, decrease; ↔, no changes; SBP: systolic blood pressure; DBP: diastolic blood pressure; LV: left ventricle; LVM: left ventricular mass; EDV: end-diastolic volume; CO-Hb: carboxyhemoglobin; LV P-V: LV pressure-volume; ED: endothelial dysfunction; HW : BW: heart weight : body weight; ROS: reactive oxygen species; NO: nitric oxide; LVPWT: left ventricular wall thickening; FS: fractional shortening; HR: heart rate; BP: blood pressure; ET-1: endothelin 1; HIF1*α*, hypoxia inducible factor; VEGF: vascular endothelial growth factor; TGF-*β*: transforming growth factor; MMP: matrix metalloproteinases; TIMP-1: tissue inhibitors of metalloproteinase-1; LVEDD: left ventricular end-diastolic diameter; LVESD: left ventricular end-systolic diameter; E/A: E: peak velocity of early ventricular filling, A: peak velocity of transmitral flow during atrial contraction; NE: norepinephrine; p38: p38 kinase; Pp38: phosphorylated p38 kinase; ERK: extracellular-regulated kinase; PERK: phosphorylated extracellular-regulated kinase; JNK: c-Jun NH2-terminal protein kinase; MPT: mitochondrial permeability transition; [Ca2+]i: intracellular calcium; [Ca2+]m: mitochondrial calcium; VM: ventricular myocytes; CVF: collagen volume fraction; RVW: right ventricular weight; CSA: cross sectional area; Cx43: connexin 43; LA: left atria; LVSV: left ventricular systolic volume; EF: ejection fraction; OHDAH: 3-hydroxyacyl coenzyme-A dehydrogenase; LDH: lactate dehydrogenase; LDL: low-density lipoprotein; VLDL: very low-density lipoprotein; TG: triacylglycerols; HDL: high-density lipoprotein; PGC-1*α*: peroxisome proliferator-activated receptor gamma coactivator 1 alpha; PPAR-*α*: peroxisome proliferator-activated receptor alpha; DA: diastolic area; SA: systolic area; LOOHs: lipid hydroperoxides; LVH: left ventricular hypertrophy; RVW : BW: right ventricular weight : body weight; LW: lung weight; eNOS: endothelial nitric oxide synthases; iNOS: inducible NOS; IL: interleukin; TNF: tumor necrosis factor; sGC: soluble guanylate cyclase; VASP: vasodilator-stimulated phosphoprotein; pVASP: phosphorylated VASP; cGMP: cyclic guanosine monophosphate; mRNA: messenger RNA; *β*-MHC: myosin heavy chain beta; PKG: protein kinase G; PDE5: phosphodiesterase type 5 inhibitor; H2S: hydrogen sulfide; PC: protein carbonyl; NoC: no conditioning, PreC: preconditioning; PostC: postconditioning; I/R: ischemia reperfusion; MDA: malondialdehyde; Akt: RAC-alpha-serine/threonine-protein-kinase.

**Table 2:** A selective list of studies highlighting the effect of drugs on LV remodeling after CS exposure.

Model	Study/drug tested/effect^¥^	Cardiac remodeling						Ref
		Structural/functional	Inflammation	Oxidative stress	Apoptosis	Metabolic impairment	Others	
S-D rats: *40 cigs/day for 4 months*	H2S effects on LV remodeling in rats subjected to CS *Protection: ++*	↓ LVEDD and LVESD ↑ LVEF and LVFS	N/A	↓ ROS ↓ MDA levels ↑ SOD and GSH-Px activities	N/A	N/A	↓ fibrosis	[125]

S-D rats: *4 cigs/day/5 days/week for 6 weeks with MI on the 5^th^ week*	N-acetyl cysteine effects on LV remodeling post-MI in rats subjected to CS *Protection: ++*	↓ infarct size ↑ LVFS ↓ LVESD ↔ LVEDD	↓ serum levels of IL-1*α*, IL-1*β*, IL-2, IL-6, IFN-*γ* and TNF-*α* ↓ cardiac mRNA expression of IL-6, IFN-*γ*, SDF-1, TGF-*β*, and TNF-*α*	↑ cardiac mRNA of SOD, TXN ↓ cardiac mRNA of p22^phox^, Keap, and Nrf2 ↑ plasma levels of glutathione	↓ PARP	N/A	↓ mRNA of MMP-2	[78]

Wistar rats: *40 cigs/day/5 days/week for 2 months*	Effects of swimming on LV remodeling in rats subjected to CS *Protection: −*	↑ LVM, LVEDP, LVH, RVH	N/A	N/A	N/A	N/A	↓ RV and LV collagen	[126]
		(i) Impairment of myocardial inotropism						

Wistar rats: *40 cigs/day for 6 months starting at 48 hours post-MI*	Effects of *β*-carotene on LV remodeling post-MI in rats subjected to CS *Protection: −*	↔ infarct size ↑ MCA	N/A	N/A	N/A	N/A	N/A	[127]

Wistar rats: *40 mg/kg/day of tobacco smoke for 2 months*	Propranolol effects on LV remodeling in rats subjected to CS *Protection: +*	↓ LVM : BW ratio, HR	N/A	N/A	N/A	N/A	N/A	[128]

Wistar rats: *40 cigs/day for 2 months*	Lisinopril effects on LV remodeling in rats subjected to CS *Protection: +*	↓ LVESD, CSA, LVW ↑ LVEF, LVFS	↔ IFN-*γ*, TNF-*α* cardiac protein levels	N/A	N/A	N/A	↔ connexin 43 protein levels ↔ collagen amount	[28]

Wistar rats: *40 cigs/day for 2 months*	Taurine effects on LV remodeling in rats subjected to CS *Protection: −*	↑ LVWT, E/A, ↑ diastolic dysfunction	↔ IFN-*γ*, TNF-*α* cardiac protein levels	N/A	N/A	↑ LDH activity	↓ Ser16P-PLN ↓ P-PLN : PLN ratio ↔ collagen volume	[129]

Albino guinea pigs: *5 cigs/day for 2 months*	Vitamin C effects on LV remodeling in guinea pigs subjected to CS *Protection: ++*	N/A	(i) Prevents neutrophil infiltration	↓ oxidative damage	↓ apoptosis	N/A	(i) No troponin T and I serum levels	[9]
							(ii) No thrombosis	
			↓ TNF-*α*, IL-1*β* cardiac protein levels				↓ fibrosis, collagen deposition ↓ LDL, TG serum levels ↓ MMP-9, 12	

Rats: *2 months of CS exposure*	Vitamin D effects on LV remodeling in rats subjected to CS *Protection: +*	↓ LVH	↔ IFN-*γ*, TNF-*α* and IL-10	↓ LOOH ↑ SOD, catalase activities	N/A	N/A	↔ collagen deposition	[123]

Wistar rats: *40 cigs/day for 4 months*	Trimetazidine effects on LV remodeling in rats subjected to CS *Protection: ++*	↓ LVW : BW ratio ↑ LVEF, LVFS, ↓ LVEDD, LVESD	↓ IL-1*β*, IL-6, and TNF-*α* cardiac mRNAs ↓ IL-1*β*, IL-6, and TNF-*α* serum levels	↓ MDA levels ↑ SOD and GSH-Px activities	↓ apoptosis	N/A	↓ fibrosis	[60]

S-D rats: *96 cigs/day passive for 6 weeks*	L-arginine effects on LV remodeling post-I/R in rats subjected to passive CS *Protection: +*	↓ infarct size ↔ HR, SP	N/A	N/A	N/A	N/A	↔vascular reactivity ↔ bleeding time	[130]

Wistar rats: *40 cigs/day for 4 months*	H2S effects on LV remodeling in rats subjected to CS *Protection: ++*	↑ LVEF, LVFS ↓ LVEDD, LVESD	N/A	N/A	↓ apoptosis	N/A	↓ autophagy	[30]

Wistar rats: *40 cigs/day for 3 months*	3-Methyladenine effects on LV remodeling in rats subjected to CS *Protection: ++*	↓ LVEDD, LVESD ↑ LVFS, LVEF	N/A	N/A	N/A	N/A	↓ autophagy ↓ Beclin1, LC3	[93]

Wistar rats: *40 cigs/day for 4 months*	Valsartan effects on LV remodeling in rats subjected to CS *Protection: ++*	↓ LVEDD, LVESD ↑ LVFS, LVEF	↓ hs-CRP, IL-6, TNF-*α* and MCP-1 serum levels	↓ MDA ↑ SOD and GSH-Px activities	↓ apoptosis	N/A	N/A	[76]

Rabbits: *6 cigs/day passive or 3 weeks*	Captopril effects on LV mitochondria in rabbits subjected to passive CS *Protection: +*	N/A	N/A	N/A	N/A	(i) Prevents Mito coQ10 decrease	N/A	[26]
						(ii) Improves OXPHOS		
						↑ F1-ATPase levels ↑ cytooxidase activity		

Rats: *20 cigs/day first week then 40 cig/day for two months*	Pentoxifylline effects on LV remodeling in rats subjected to CS *Protection: +*	↓ LA area ↑ LV systolic function	↔ IL-10, ICAM-1, TNF-*α*	↑ SOD and GSHPx activities	↔ caspase-3	↓ LDH, CS, 3-OH-DHA	N/A	[90]

Wistar rats: *40 cigs/day for two months*	Spironolactone effects on LV remodeling in rats subjected to CS *Protection: 0*	↔ cardiac hemodynamics and structure	↔ IFN-*γ*, TNF-*α*, IL-10, ICAM-1	N/A	N/A	N/A	↔ GLUT4 ↔ collagen volume	[79]

^¥^Results presented in this table are in comparison with nonsmoking same conditioning treatment. CS: chronic tobacco smoking; N/A: not available; ↑, increase; ↓, decrease; ↔, no changes; H2S: hydrogen sulfide; LV: left ventricle; LVEDD: left ventricular end-diastolic diameter; LVESD: left ventricular end-systolic diameter; LVEF: left venticular ejection fraction; LVFS: left ventricular fractional shortening; LVEDP: left ventricular diastolic pressure; LVWT: left ventricular wall thickness; LVH: left ventricular hypertrophy; RVH: right ventricular hypertrophy; LDL: low-density lipoprotein; TG: triacylglycerols; SOD: superoxide dismutase; GSH-Px: glutathione peroxidase; MDA: malondialdehyde; MI: myocardial infarction; IL: interleukin; IFN: interferon; TNF: tumor necrosis factor; SDF-1: stromal cell-derived factor 1; TGF: transforming growth factor; TXN: thioredoxin; mRNA: messenger RNA; p22^phox^, cytochrome *b*_558_* α*-subunit; Keap: Kelch-like ECH-associated protein 1; Nrf2: nuclear factor (erythroid-derived 2)-like 2; PARP: poly [ADP-ribose] polymerase 1; MMP: matrix metalloproteinases; RV: right ventricle; LVM : BW: left ventricular mass : body weight; HR: heart rate; LVW: left ventricular weight; CSA: cross-sectional area; LDH: lactate dehydrogenase; Ser16 P-PLN: serine 16 phosphorylated phospholamban; P-PLN : PLN: phosphorylated phosfolamban/phospholamban ratio; E/A: E: peak velocity of early ventricular filling, A, peak velocity of transmitral flow during atrial contraction; Mito coQ10: mitochondrial coenzyme Q_10_; LC3: microtubule-associated protein 1A/1B-light chain 3; hs-CRP: C-reactive protein; MCP-1: monocyte chemotactic protein-1; OXPHOS: oxidative phosphorylation chain; LA: left atria; ICAM-1: intercellular adhesion molecule 1; 3-OH-DHA: 3-hydroxyacyl coenzyme A dehydrogenases; GLUT4: glucose transporter type 4.

## Abbreviations

CVDs: Cardiovascular diseases
IL: Interleukin
LV: Left ventricle
MI: Myocardial Infarction
MMPs: Matrix metalloproteinases
CS: Chronic tobacco smoking
CAD: Coronary artery disease
LVEDD: Left ventricular end-diastolic diameter
LVESD: Left ventricular end-systolic diameter
FS: Fractional shortening
RIMD: Oxidative stress-inflammation-metabolic impairment-cell death
MMP: Matrix metalloproteinase
MAPK: Mitogen-activated protein kinase
ROS: Reactive oxygen species
GSH-px: Glutathione peroxidase
SOD: Superoxide dismutase
FA: Fatty acid
GSSG: Glutathione disulfide
GSH: Glutathione
MAPKs: Mitogen-activated protein kinases
NF-κB: Nuclear factor-κB
TNF-*α*: Tumor necrosis factor-alpha
coQ10: Coenzyme 10
ERK1/2: Extracellular signal-regulated kinase
CABG: Coronary artery bypass grafting
PCI: Percutaneous coronary intervention.
